# MicroRNA-29a Counteracts Synovitis in Knee Osteoarthritis Pathogenesis by Targeting VEGF

**DOI:** 10.1038/s41598-017-03616-w

**Published:** 2017-06-15

**Authors:** Jih-Yang Ko, Mel S. Lee, Wei-Shiung Lian, Wen-Tsan Weng, Yi-Chih Sun, Yu-Shan Chen, Feng-Sheng Wang

**Affiliations:** 1 Departments of Orthopedic Surgery, Chang Gung University College of Medicine, Kaohsiung Chang Gung Memorial Hospital, Kaohsiung, Taiwan; 2 Core Laboratory for Phenomics & Diagnostics, Chang Gung University College of Medicine, Kaohsiung Chang Gung Memorial Hospital, Kaohsiung, Taiwan; 3 Department of Medical Research, Chang Gung University College of Medicine, Kaohsiung Chang Gung Memorial Hospital, Kaohsiung, Taiwan; 4 Graduate Institute of Clinical Medical Sciences, Chang Gung University College of Medicine, Kaohsiung Chang Gung Memorial Hospital, Kaohsiung, Taiwan

## Abstract

Synovitis contributes to the development of osteoarthritis (OA) of the knee. MicroRNAs regulate joint microenvironment homeostasis and deterioration. This study was undertaken to characterize the actions of microRNA-29a (miR-29a) to synovial remodeling in OA joints. Synovial specimens isolated from patients with end-stage OA knees showed abundant fibrotic matrix and vessel histopathology concomitant with weak miR-29a expression. *In vitro*, miR-29a knockdown caused synovial fibroblasts to exhibit high expressions of collagen III, TGF-β1, MMP9, MMP13, and ADAMTS5, whereas miR-29a overexpression diminished these joint-deleterious factors. In collagenase-mediated OA pathogenesis, miR-29a-overexpressing transgenic mice showed minor responses to hyperplasia, macrophage infiltration, fibrosis, hyperangiogenesis, and VEGF expression in synovial lesions. These effects mitigated articular cartilage loss and gait aberrance of injured joints. Intra-articular administration of miR-29a precursor lessened the collagenase aggravation of excessive synovial remodeling reactions and thereby sustained joint tissue integrity. miR-29a lowered VEGF production and angiogenic activities in synovial fibroblasts through targeting the 3′-UTR of VEGF. Taken together, miR-29a deficiency exacerbated synovitis pathogenesis in the end-stage OA knees. miR-29a signaling fends off excessive synovial angiogenesis and fibrosis, which delays joint destruction. This study sheds new light on the protective effects against synovial deterioration and the therapeutic advantage of miR-29a in OA knees.

## Introduction

Synovial microenvironment deterioration results in joint integrity loss, which prompts the development of osteoarthritis (OA)^1^. Excessive synovial fibrosis is linked to the occurrence of joint destabilization and stiffness in knee OA progression^2, 3^. The hyperangiogenic and inflammatory synovial compartment is found to accumulate overabundant fibrotic matrices^4^ and secrete proteolytic factors^5, 6^ that disintegrate cartilage and synovial homeostasis^7^ within affected joints. The molecular mechanism underlying excessive fibrosis reactions in the synovium warrants investigation.

MicroRNAs constitute 18−25 nucleotides that participate in tissue development and pathogenesis through disrupting the mRNA expression of the target^8^. An increasing number of studies show the involvement of microRNAs in the pathogenesis of OA joints^9^. For example, serum let-7 levels are correlated with the incidence of severe knee and hip OA^10^. miR-602 and miR-608 regulate MMP13 expression^11^; miR-30 reduces the ADAMTS-5 levels in OA cartilage chondrocytes^12^. miR-146a mediates the denbinobin reduction of vascular cell adhesion molecule-1 expression in OA synovial fibroblasts^13^. miR-210 signaling is involved in the connective tissue growth factor-mediated angiogenic activities of OA synovial fibroblasts^14^. Treatment with exogenous miR-146a and miR-183 mimics alleviates joint pain in rats with medial meniscus transection-mediated knees^15^.

Of microRNAs, the miR-29 family is observed to modulate angiogenic reactions, immune responses, and metabolic activities in various tissues^16, 17^. miR-29 deficiency worsens the pathogenesis of fibrotic matrix accumulation in the bleomycin induction of systemic sclerosis^18^. It also regulates carbon tetrachloride^19^- and bile duct ligation-injured liver fibrosis^20^. Gain of miR-29a signaling attenuates the diabetes elevation of fibrogenic activity within the kidney microenvironment^21^ and the asphyxiation exaggeration of excessive extracellular matrix synthesis in pulmonary tissues^22^. In addition to fibrosis reactions, the miR-29 family modulates chondrocyte metabolism in OA articular cartilage^23^. Levels of miR-29c-3p in synovial fluid are associated with the severity of OA knees^24^. The contribution of miR-29 signaling to synovial fibrosis in OA knees remains elusive.

This study was undertaken to analyze the association of miR-29 expression and synovial fibrosis within end-stage OA knees and investigate the fibrogenic and angiogenic activities of synovial fibroblasts in response to miR-29a signaling. Using miR-29a transgenic mice and exogenous miR-29a treatment, we also characterized the synovial integrity, articular cartilage morphology, and gait profiles during collagenase-mediated OA knee pathogenesis.

## Results

### Low miR-29a expression was associated with synovial fibrosis in OA knees

**Figure 1:**
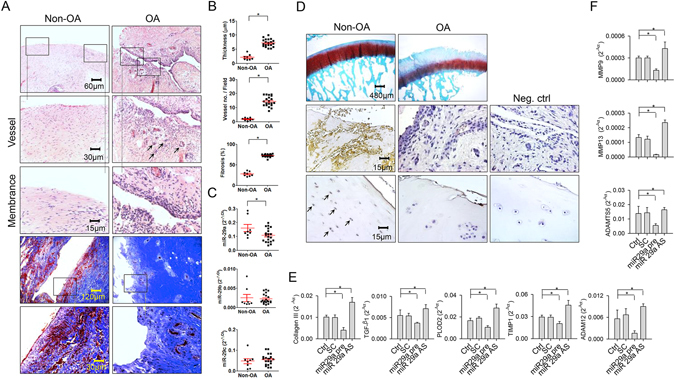
Analyses of fibrotic reactions in end-stage OA synovial tissues and fibroblast cultures. (**A**) Histochemical images of synovial hyperplasia, hyperangiogenesis (arrows), and fibrotic tissue positive for Masson’s trichrome stain in the end-stage OA group. Panels in the low power field images indicate areas of interest for high power field images. (**B**) OA group showed increases in synovial membrane thickness, vessel formation, and fibrosis tissue area. (**C**) OA synovial tissues exhibited high miR-29a but not b, c expression. (**D**) The OA cartilage displayed weak Safranin-O staining. Synovial fibroblasts and chondrocytes (arrows) in the OA group displayed weak miR-29a transcripts. Data (mean ± standard error) are calculated from 20 patient with end-stage OA and 8 participants with non-OA injury and analyzed by a Wilcoxon test. miR-29a transfection reduced expression of (**E**) collagen III, TGF-β1, PLOD2, TIMP1, and ADAM12 in concomitant with (**F**) low expression of MMP9, MMP13 and ADAMTS5 in synovial fibroblasts. Loss of miR-29a signaling increased fibrogenic factor and proteinase expression in cell cultures. Synovial fibroblast experiments in triplicate were repeated 3 times. All investigations (mean ± standard error) of synovial fibroblasts were analyzed by a parametric analysis of variance (ANOVA) and a Bonferroni post-hoc test. Asterisks (*) stands for significant difference between groups.

We investigated whether miR-29 expression was relevant to the occurrence of synovial fibrosis within end-stage OA knees. Synovial tissues in the OA group exhibited hyperplasia concomitant with intensive micro-capillary vessel morphology (Fig. 1A). Masson’s trichrome histochemical staining revealed that the OA synovial membrane predominantly displayed dense fibrogenic matrix collagen (blue), whereas distinguishable non-fibrosis tissue (red) remained within the non-OA specimens (Fig. 1A). The OA group showed significant increases in synovial membrane thickness, vessel number, and fibrosis tissue area (Fig. 1B). In addition to the fibrosis histopathology, expression of miR-29a instead of miR-29b and miR-29c in the OA group was significantly lower than that in the non-OA group (Fig. 1C). Affected articular tissues in the OA group exhibited evident cartilage destruction as evidenced by weak Safranin O stain. Consistent with RT-quantitative PCR analyses, synovial fibroblasts and chondrocytes in affected joints in the OA group expressed slight miR-29a transcripts as probed by *in situ* hybridization (Fig. 1D).

Experiments were carried out to test if miR-29a altered fibrogenic reactions in synovial fibroblasts. Collagen III, TGF-β1, TIMP1, PLOD2, and ADMS12 are observed to contribute to fibrotic matrix accumulation in synovial fibroblasts in OA joints^2^. RT-quantitative PCR analyses uncovered that cell cultures transfected with miR-29a precursor showed significant reductions in collagen III, TGF-β1, PLOD2, TIMP1, and ADMS12 expression (Fig. 1E). They also displayed remarkable declines in cartilage degradation factors MMP9, MMP13, and ADAMT5 expression (Fig. 1F). On the contrary, miR-29a antisense oligonucleotide transfection distinguishably increased expression of fibrogenic factors (Fig. 1E) and proteinases (Fig. 1F). Scramble control transfection did not significantly affect the expression of cartilage-deleterious factors in cell cultures compared to those in the control.

### miR-29a shielded from synovial deterioration

**Figure 2:**
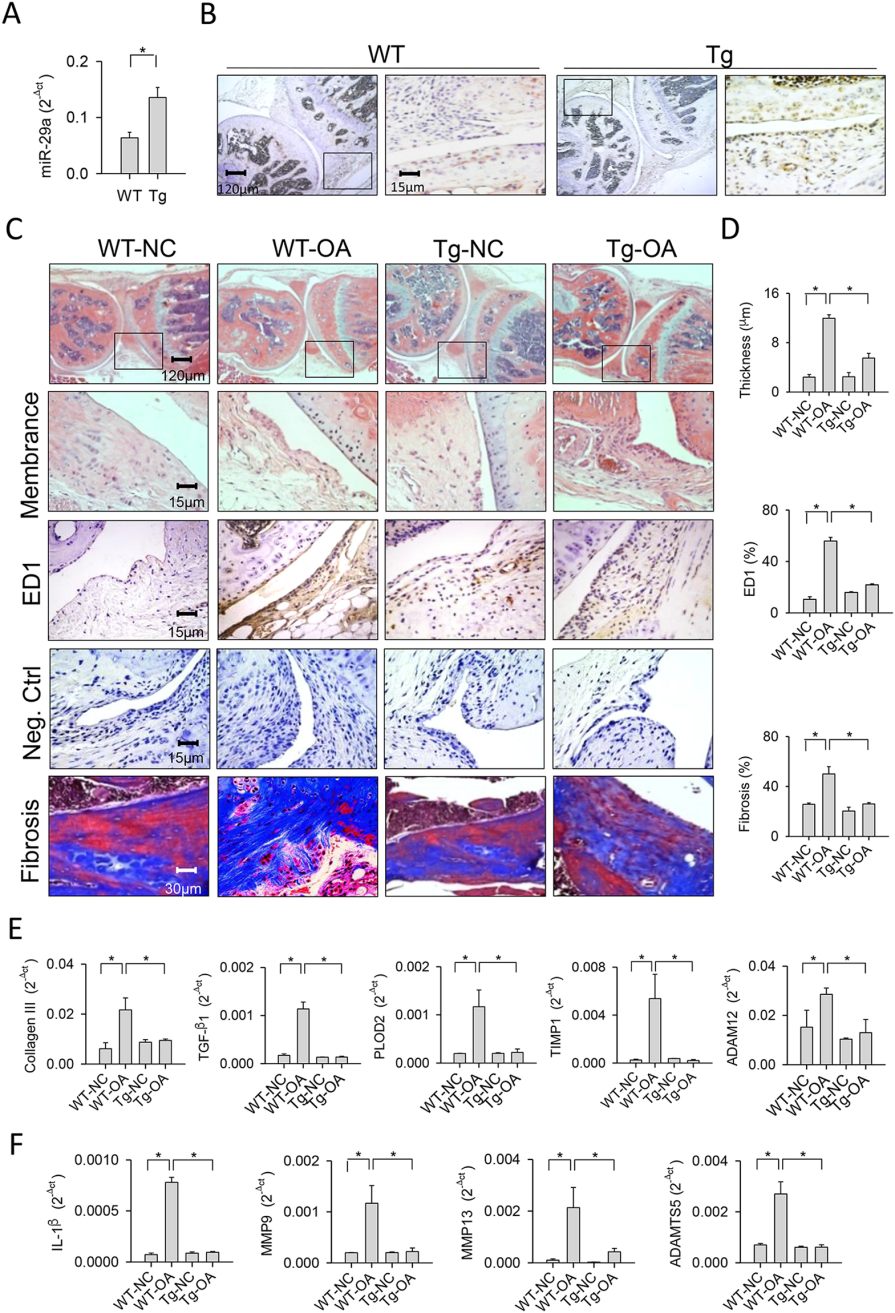
Analyses of synovial tissues in collagenase-affected joints in the miR-29aTg mice and wild-type mice. (**A**) Synovial tissues in the miR-29aTg mice showed high miR-29a expression probed by RT-qPCR and analyzed using a Wilcoxon test. (**B**) They also displayed strong miR-29a transcripts as evidenced by *in situ* hybridization. Panels in the low power field images indicate areas of interest for high power field images of synovial membrane. (**C**) Synovial tissues in the miR-29aTg mice exhibited slight hypertrophy, ED1-positive macrophage infiltration, and fibrotic matrix accumulation in collagenase-injured joints. (**D**) miR-29Tg mice had minor responses to collagenase aggravation of membrane thickness, macrophage number, and fibrosis tissue area. miR-29a overexpression reduced the collagenases enhancement of (**E**) collagen III, TGF-β1, PLOD2, TIMP1, ADAM12, (**F**) IL-1β, MMP9, MMP13, and ADAMTS5 expressions within injured joints. Each bar plot stands for mean ± standard error calculated from 8 animals in each group and analyzed by a parametric analysis of variance (ANOVA) and a Bonferroni post-hoc test. Asterisks (*) stands for significant difference between groups.

Given that miR-29a signal lowered the expression of joint-deleterious factors in synovial fibroblasts, we used miR-29a-overexpressing transgenic mice (miR-29aTg) and verified whether miR-29a affected joint integrity in the collagenase-mediated OA pathogenesis. The miR-29Tg mice exhibited a significant increase in miR-29a expression in synovial tissue (Fig. 2A). Fibroblasts adjacent to the synovium compartment in the miR-29aTg mice showed strong miR-29a transcripts (Fig. 2B). Body weight, serum biochemistry and feed intake of the miR-29aTg mice were comparable with those of the littermate wild-type mice that did not bear the construct (data not shown). These miR-29aTg mice have been found to show minor responses to bile duct ligation-induced hepatic fibrosis^20^ and hyperglycemia exaggeration of renal fibrosis^21^. In wild-type mice, the synovial compartment within affected joints showed distinguishable hyperplasia and hypercelluarity. A great number of macrophages that were positive for ED1 immunostaining and abundant Masson’s trichrome stain-positive fibrotic matrix existed within the lesion site (Fig. 2C). In the miR-29aTg mice, synovial tissue exhibited slight thickening, macrophage filtration, and fibrosis in the collagenase-injured joints (Fig. 2C). Affected knees in the wild-type mice also showed significant increases in membrane thickness, number of ED1-immunostained macrophages, and fibrotic tissue area of the synovial compartment. These adverse actions to synovial histomorphometry were evidently mitigated in the miR-29aTg mice (Fig. 2D). In addition to histology, there were remarkable increases in collagen III, TGF-β1, PLOD2, TIMP1, and ADAM12 expressions in affected joints in the wild-type mice (Fig. 2E). IL-1β, ADAMTS5, MMP9, MMP13 expressions within injured knees of the wild-type mice were also significantly elevated (Fig. 2F). These escalating effects on fibrogenic factor (Fig. 2E), proteinase, and inflammatory cytokine expressions (Fig. 2F) were remarkably weakened in the miR-29aTg mice.

### miR-29a attenuated angiogenic reactions in synovial compartment

**Figure 3:**
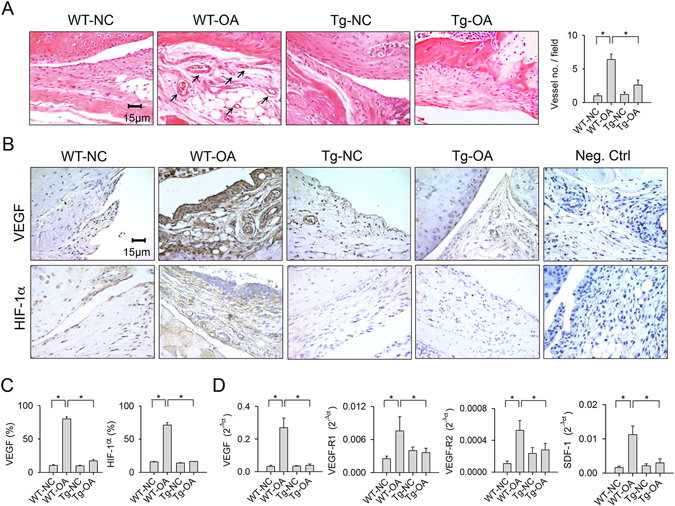
Analyses of angiogenic activities in synovial tissues. (**A**) Few vessels (arrows) were developed within collagenase-injured joints in the miR-29aTg mice. (**B**) Synovial tissues in the miR-29aTg mice displayed minor response to collagenase escalation of VEGF and HIF-1α immunostaining. (**C**) miR-29a overexpression reduced the number of synovial fibroblasts positive for VEGF and HIF-1α. (**D**) miR-29a reduced the collagenase elevation of VEGF, VEGF-R1, VEGF-R2, and SDF-1 expression in synovial tissues. Each bar plot stands for mean ± standard error calculated from 8 animals in each group and analyzed by a parametric analysis of variance (ANOVA) and a Bonferroni post-hoc test. Asterisks (*) stands for significant difference between groups.

Excessive angiogenesis is a prominent feature of synovial inflammation and remodeling during OA progression^5^. Histological investigations revealed that affected synovium in the wild-type mice showed abundant vessel formation in conjunction with a remarkable increase in the number of vessels (Fig. 3A). Synovial cells and vessels adjacent to affected synovium within the wild-type mice exhibited strong VEGF and angiogenic transcription factor HIF-1α immunostaining, whereas synovium in the collagenase-treated miR-29aTg mice showed weak immunoreactivity (Fig. 3B). Synovial lesions caused by collagenase in the wild-type mice displayed significant increases in the number of VEGF- and HIF-1α-immunostained cells. These effects were evidently mitigated in the miR-29aTg mice (Fig. 3C). In addition, affected synovial tissue in the wild-type mice displayed significant increases in expressions of angiogenic regulators VEGF, VEGF-R1, VEGF-R2, and SDF-1. The miR-29aTg mice showed minor responses to the collagenase provocation of agniogenic factor expression in synovial compartment (Fig. 3D).

### miR-29a lessened the collagenase impediment of cartilage integrity and gait property

**Figure 4:**
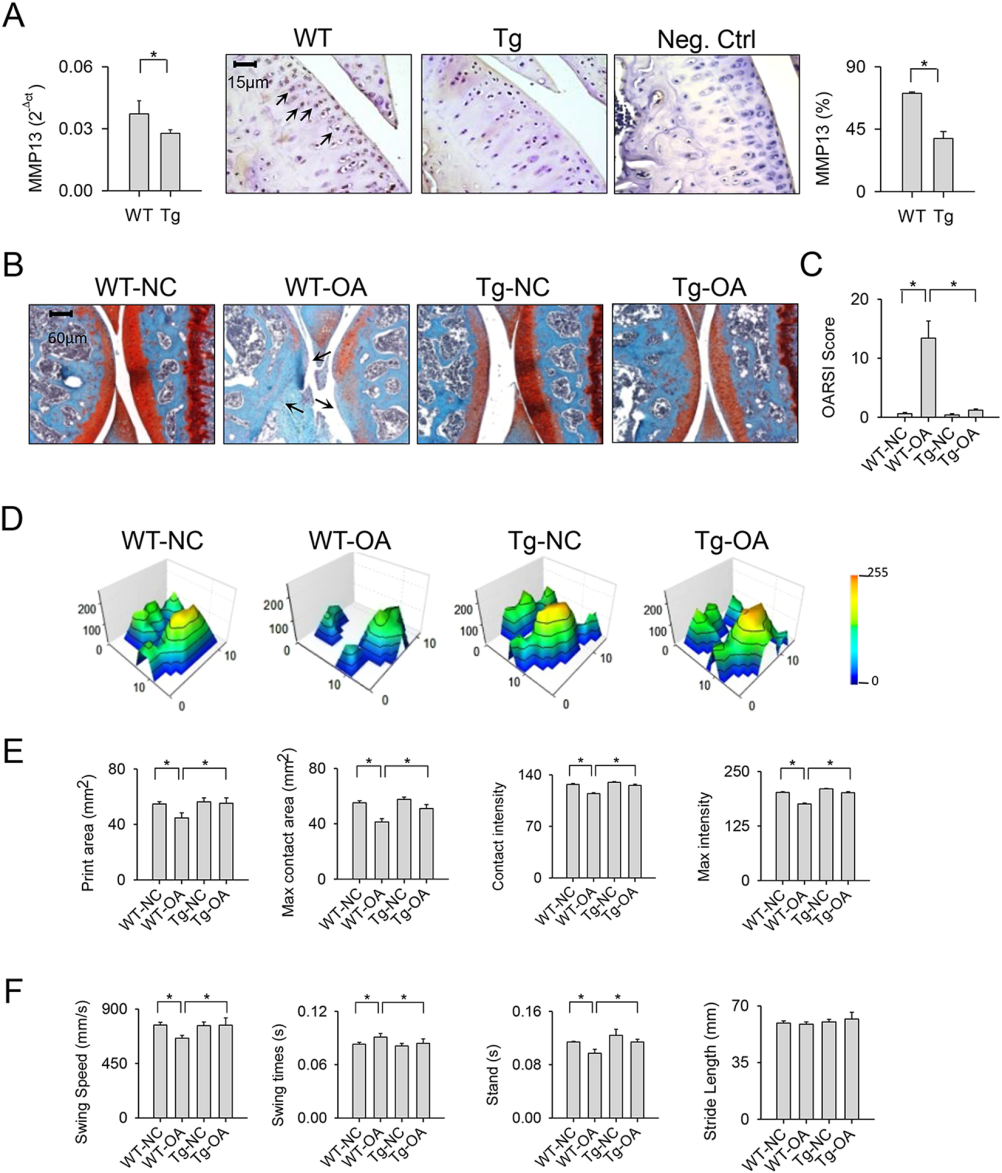
Cartilage morphology and gait characteristics of collagenase-affected joints in miR-29aTg mice and wild-type mice. (**A**) Articular chondrocytes in the miR-29aTg mice displayed weak MMP13 mRNA expression and immunostaining (arrows) in knee joints. (**B**) Injured knees in the miR-29aTg mice showed smooth articular cartilage morphology (arrows) and intense Safarin O staining in association with (**C**) lower OARSI scores than those of the wild-type mice. (**D**) The miR-29aTg mice showed regular footprint histograms in the collagenase-injected knees. The miR-29aTg mice exhibited minor response to the collagenase disturbance of (**E**) print area, maximum contact area, contact intensity, maximum intensity affected joints. (**F**) The collagenase disturbance of wing speed, swing time, and stand time were lessened in the miR-29aTg mice. Each bar plot stands for mean ± standard error calculated from 8 animals in each group and analyzed by a parametric analysis of variance (ANOVA) and a Bonferroni post-hoc test. Asterisks (*) stands for significant difference between groups.

Regarding cartilage morphology, primary chondrocytes isolated from articular cartilage in the miR-29aTg mice exhibited a significant reduction in MMP13 mRNA expression. Immunohistochemistry confirmed that chondrocytes in the articular cartilage showed weak MMP-13 immunoreactivity (Fig. 4A). The collagenase-injured joints in the wild-type mice displayed articular cartilage loss in association with slight safranin-O histochemical staining. The affected joints in the miR-29aTg mice showed smooth articular morphology and intense cartilage matrix microstructure (Fig. 4B). Affected joints in the wild-type mice exhibited a significant increase in OARSI scores. The extent of cartilage damage was distinguishably weakened in the miR-29aTg mice (Fig. 4C).

In addition to joint histology, the wild-type mice with injured joints walked in a rigid way and showed tiptoed footprint histograms as compared with the control group (Fig. 4D). All the miR-29aTg mice walked briskly and exhibited steady footprints (Fig. 4D). Consistent with the walking patterns, the collagenase-treated wild-type mice displayed significant reductions in print area, maximum intensity, contact area, and contact intensity of the paws (Fig. 4E). Affected joints also showed lower swing speed and stand time in conjunction with a higher swing time as compared with the control group (Fig. 4F). These impairments of footprint (Fig. 4E) and gait characteristics (Fig. 4F) were remarkably weakened in the collagenase-treated miR-29aTg mice. However, the stride length of injured knees in the wild-type and miR-29aTg mice was not significantly changed after collagenase injection (Fig. 4F).

### Exogenous miR-29a treatment improved synovitis in affected knees

**Figure 5:**
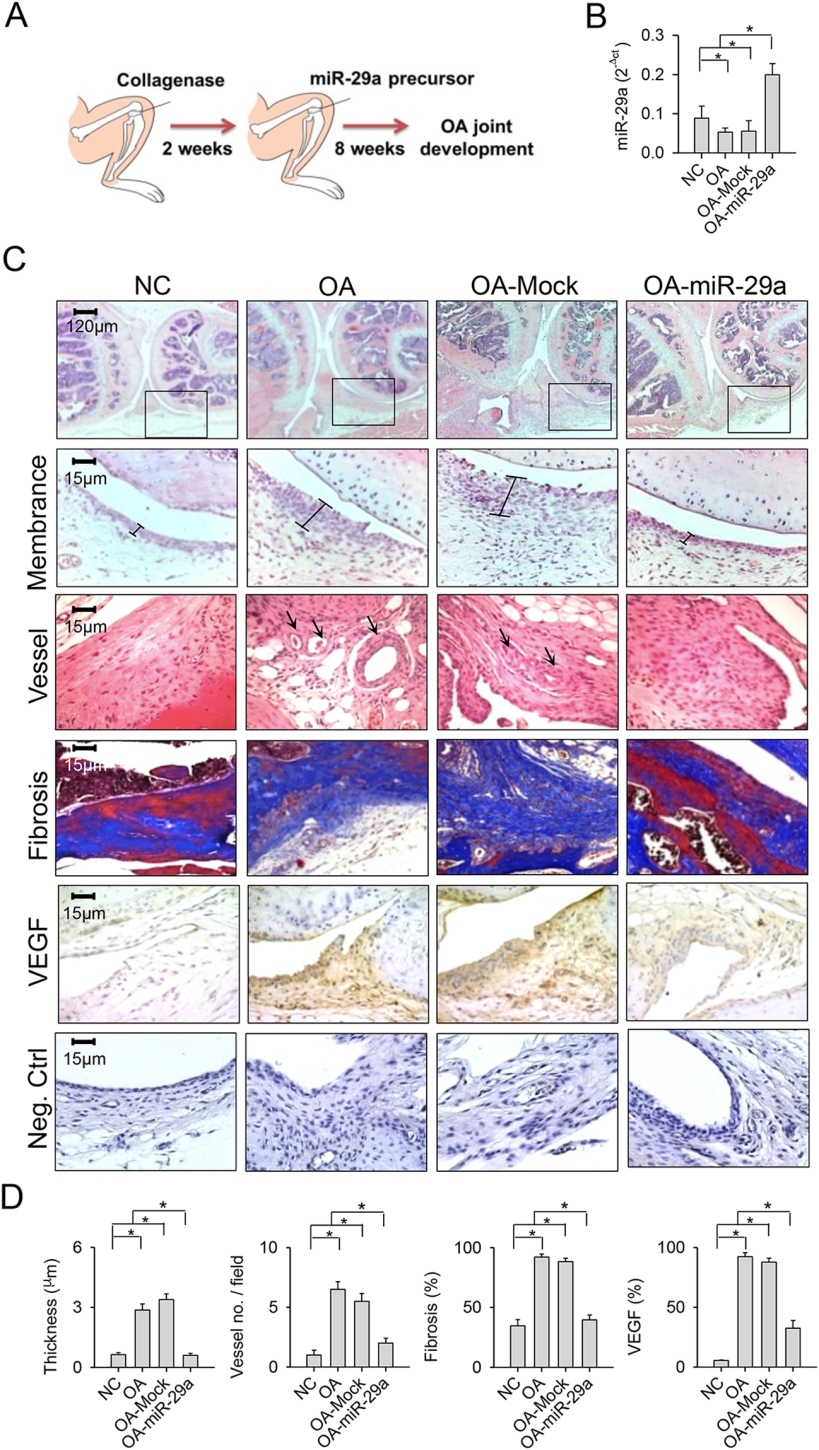
Effects of exogenous miR-29a administration on synovial histology. (**A**) Scheme of miR-29a administration of collagenase-affected knees. (**B**) Synovial tissues exhibited high miR-29a expression after treatment. (**C**) The treatment attenuated cartilage damage, hyperangiogenesis (arrows), fibrosis, and VEGF immunostaining in collagenase-affected synovial tissue. Panels in the lower power field images indicate area of interest for the high power field images of synovial membrane. (**D**) Membrane thickness, vessel number, fibrosis area, and VEGF expression within affected synovium were lessened after miR-29a treatment. Each bar plot stands for mean ± standard error calculated from 8 animals in each group and analyzed by a parametric analysis of variance (ANOVA) and a Bonferroni post-hoc test. Asterisks (*) stands for significant difference between groups.

We tested whether miR-29a administration affected synovium integrity during OA knee development. At 2 weeks after collagenase injection, lentivirus-shuttled miR-29a precursor was administered via intra-articular injection (Fig. 5A). This treatment enabled synovial tissues to maintain a significant elevation in miR-29a expression at 8 weeks after collagenase stress. Mock treatment did not significantly affect miR-29a expression in the injured joints (Fig. 5B). Affected joints in the miR-29a-treated group showed slight membrane thickening, fibrotic matrix deposition concomitant with minor neovessel formation and VEGF immunostaining within the synovial compartment (Fig. 5C). Consistent with the changes in synovium morphology, the extent of collagenase exaggeration of membrane thickness, fibrotic tissue area, and number of vessels and VEGF-immunostained cells was distinguishably weakened after exogenous miR-29a administration (Fig. 5D).

**Figure 6:**
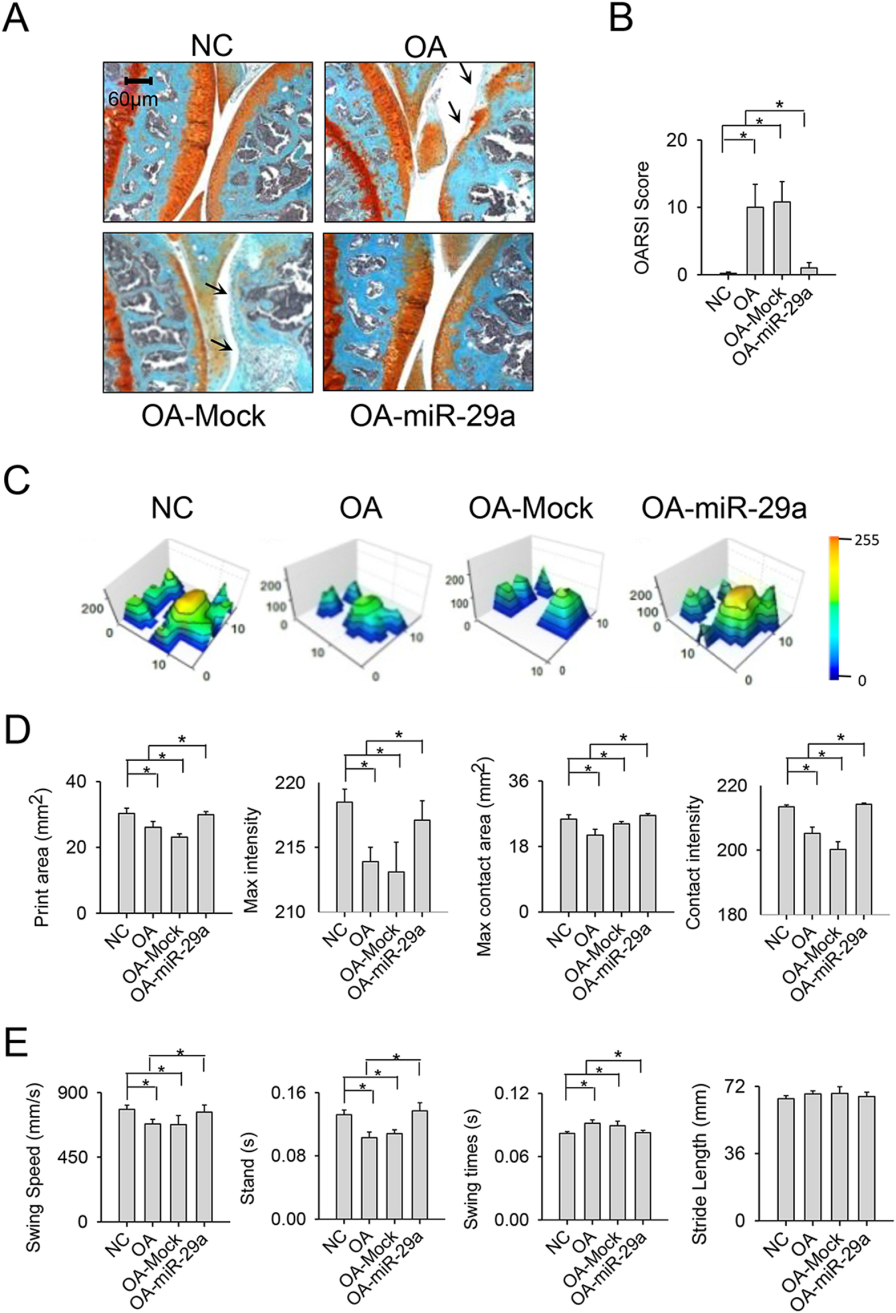
Effects of exogenous miR-29a administration on gait characteristic and cartilage morphology. The miR-29a-administered affected joints exhibited (**A**) smooth articular cartilage morphology (arrow) concomitant with (**B**) low OARSI scores. (**C**) The miR-29a-treated knees showed regular footprint histograms. miR-29a treatment ameliorated the collagenase deterioration of (**D**) print area, maximum contact area, contact intensity, maximum intensity affected joints. (**E**) The collagenase aberration of swing speed, swing time, and stand time of injured knees was mitigated after the miR-29a treatment. Each bar plot stands for mean ± standard error calculated from 8 animals in each group and analyzed by a parametric analysis of variance (ANOVA) and a Bonferroni post-hoc test. Asterisks (*) stands for significant difference between groups.

Of note, affected joints in the miR-29a-treated group exhibited regular cartilage morphology and intense Safranin-O staining, whereas the OA group showed severe cartilage fragmentation and microstructure loss and slight Safarin O staining (Fig. 6A). The miR-29a-treated injured knees displayed less OARSI scores than those in the OA group (Fig. 6B). Likewise, the treatment ameliorated the collagenase-mediated disturbance of footprint histogram (Fig. 6C) and significantly improved the print area, maximum intensity, maximum contact area, contact intensity of paws (Fig. 6D), swing speed, swing and stand time of injured joints of injured knees (Fig. 6E). Mock treatment did not evidently change the collagenase aggravation of synovial vascularization, fibrosis, cartilage deterioration or gait profiles in the joint microenvironment.

### miR-29a targeted VEGF expression and angiogenic activity in synovial fibroblasts

**Figure 7:**
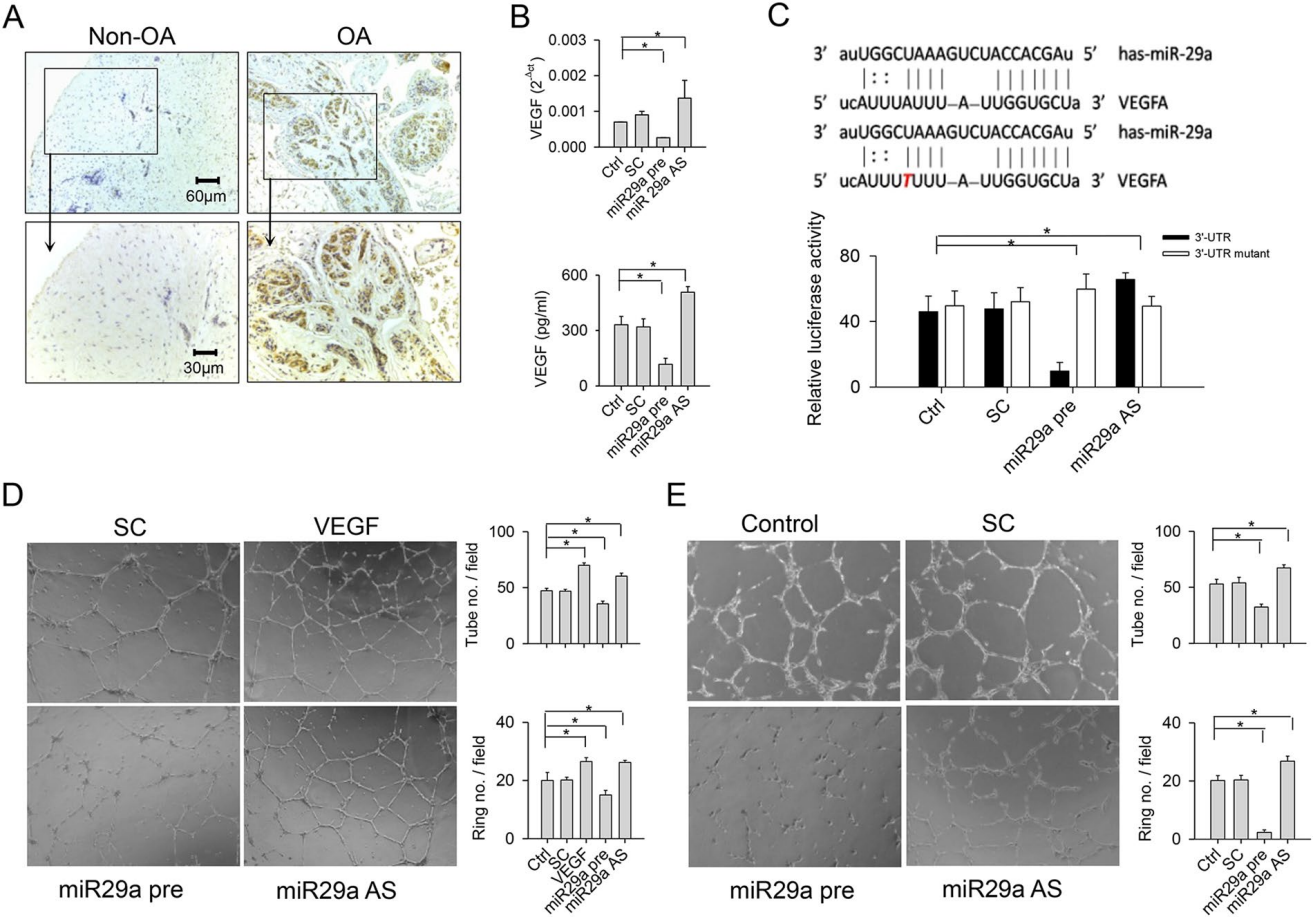
Analyses of miR-29a action to VEGF expression in synovial fibroblasts. (**A**) Synovial cells expressed strong VEGF immunostaining in synovial specimens from patients with end-stage OA knees. (**B**) miR-29a transfection reduced VEGF mRNA expression of synoival fibroblasts and VEGF levels in culture supernatants. miR-29a antisense oligonucleotide transfection increased VEGF expression. (**C**) The sequence of miR-29a binding to the intact and mutated 3′-UTR of VEGF. miR-29a precursor reduced the VEGF 3′-UTR luciferase reporter activity. miR-29a antisense oligonucleotide increased luminescence reaction. (**D**) HUVECs exposed to culture supernatants from the miR-29a-transfected synovial fibroblasts reduced tube and vessel ring formation. (**E**) HUVECs transfected with miR-29a showed low angiogenesis. Each bar plot stands for mean ± standard error calculated from at least 4 times in each group and analyzed by a parametric analysis of variance (ANOVA) and a Bonferroni post-hoc test. Asterisks (*) stands for significant difference between groups.

Bioinformatics indicates that VEGF mRNA expression is putatively targeted by miR-29a through binding of its 3′-UTR (www.microrna.org). Given that miR-29a weakened hyperangiogenesis during synovitis, we tested whether miR-29a had a direct action to angiogenic factor VEGF expression in synovial fibroblasts isolated from the end-stage OA group. Immunohistochemical analyses confirmed that synovial specimens of the OA group displayed strong VEGF immunostaining compared with the non-OA group (Fig. 7A). Gain of miR-29a signaling led to synovial fibroblasts displaying significant decreases in VEGF mRNA expression and protein secretion in culture supernatants (Fig. 7B). On the contrary, knockdown of miR-29a resulted in remarkable increases in VEGF expression (Fig. 7B).

Analyses of the VEGF 3′-UTR luciferase reporter revealed that luminescence reactions in synovial fibroblasts were significantly reduced after miR-29a transfection, whereas they were remarkably increased in the cell cultures transfected with miR-29a antisense oligonucleotide (Fig. 7C). miR-29a did not distinguishably change the luminescence reactions in cell cultures transfected with a mutated VEGF 3′-UTR luciferase reporter (Fig. 7C).

We examined whether miR-29a signaling affected the *in vitro* angiogenic activities of synovial fibroblasts. Human umbilical vessel endothelial cells (HUVECs) were incubated in media containing culture supernatants from miR-29a precursor- and antisense oligonucleotide-transfected synovial fibroblasts. HUVECs responded to VEGF recombinant protein by exhibiting intensive tube and vessel ring formation. Declines in tube and vessel ring formation in the miR-29a-transfected group were observed, whereas the miR-29a antisense oligonucleotide-transfected group showed significant elevations in angiogenic reactions (Fig. 7D). Likewise, HUVECs transfected with lentivirus-shuttled miR-29a showed remarkable decreases in tube and vessel ring morphogenesis, while they displayed significant increases in angiogenic capacity after transfection with miR-29a antisense oligonucleotide. Scrambled control treatment did not significantly affect angiogenesis of HUVECs in comparison with the control group (Fig. 7E).

## Discussion

Synovial fibrosis is a prominently deleterious reaction linked to the prevalence of symptomatic pain^25^ and biomechanical dysfunction^26^ of affected joints in the pathogenesis of OA. Accumulating studies uncover the involvement of several bio-active molecules that modulate autophagic reactions^27^, fat metabolism^28^, inflammatory activities^29^, and extracellular matrix biosynthesis^30^ in the maintenance of synovial microenvironment integrity during OA development. The mechanistic underlying microRNA regulation of extracellular matrix overproduction in OA synovial tissue is worthy of characterization. The current study is the first indication to reveal an association of miR-29a expression with the occurrence of synovial fibrosis in OA knees. It also offers new insights into the biological function and remedial potential of miR-29a in terms of facilitating synovial compartment homeostasis, rewinding OA joint progression.

Analyses of clinical specimens revealed the concomitant occurrence of miR-29 deficiency and intensive synovial lining hypertrophy and fibrosis within end-stage OA knees. Many catabolic activities occur in OA synovial microenvironments. For example, synovial tissues respond to deleterious stress by provoking synovial thickening, inflammatory cell infiltration, and angiogenesis, which subsequently result in fibrotic matrix overproduction^31^. Experimental results of synovial fibroblast cultures showed that miR-29a precursor caused cell cultures to show low expressions of TGF-β1 and collagen III, which are important regulators involved in fibrotic matrix synthesis in the synovium^2, 32^. High fibrogenic activity in cell cultures after miR-29a knockdown also extrapolated the existence of abundant fibrotic matrices in synovial compartment in end-stage OA. Our analyses agreed with other studies demonstrating that the miR-29 family reduces TGF-β1 signaling exaggeration of fibrotic matrix deposition in fibroblast cultures from cardiac^33^, lung^34^, and systemic sclerotic tissues^35^. Current investigations indicated that miR-29a signaling exerted a protective function against excessive synovial remodeling. They also captured our attention in terms of rationalization of the use of miR-29aTg mice to verify whether gain of miR-29a function affected synovial microenvironment homeostasis during OA development.

Footprint aberrance of collagenase-injured joints was notably diminished in the miR-29aTg mice. Stabilization of the synovial compartment within OA joints is found to reduce posture imbalance and gait irregularity during movement^36, 37^. Plausible investigations were that miR-29aTg mice displayed slight responses to synovial thickening, inflammatory cell infiltration, and fibrosis. The analyses of low profibrogenic factor expression in miR-29aTg mice were in line with those of investigations of synovial fibroblasts from clinical specimens. In addition to moderate synovitis, reductions in MMP9, MMP13, and ADAMTS5 expressions in injured joints also explained the occurrence of slight cartilage lesion in the miR-29aTg mice. The biological response in the joint microenvironment to miR-29 remained inconclusive. A previous study has shown that miR-29a reduces the IL-1β promotion of MMPs and ADAMTS5 expression in human chondrocyte cultures^23^; however, miR-29 expression in destabilized medial meniscus-induced joint injury and synovial explants exposed to IL-1β stress is distinguishably promoted^24^. Our study revealed the first investigation of miR-29a maintenance of synovial homeostasis that delayed OA progression at 8 weeks after collagenase injection. It is speculated that different OA models and joint-detrimental factor stresses may have different miR-29a functions. The miR-29a orchestration of synovial and cartilage metabolism during OA warrants further characterization.

Hyperangiogenesis contributes to synovial inflammation and microstructure deterioration in the processes of OA^38^ and trauma-mediated joint injury^39^. Lesions in the miR-29aTg mice had minor response to collagenase-induced hypervascularity in conjunction with low expressions of VEGF, VEGF receptors, and HIF-1α. Of the angiogenic factors, VEGF was targeted by miR-29a through interaction with its 3′-UTR, which led to reduce VEGF secretion of synovial fibroblasts. Inhibition of angiogenesis in the joint microenvironment is found to delay OA progression^40^. In this study, the low tube formation capacity in endothelial cell cultures also explained the miR-29a inhibition of angiogenic capacity of synovial fibroblasts. These findings shed a new light on the mechanistic event underlying angiogenesis deregulation of synovial integrity in OA joints.

VEGF signaling augmentation is found to exacerbate joint OA formation^41^. In view of the slight synovitis in the miR-29aTg mice, exogenous miR-29a treatment was postulated to have protective effects against excessive synovial remodeling during OA progression. Consistent with the analyses of the miR-29a transgenic mice, intra-articular administration of miR-29a precursor caused injured knees to exhibit minor VEGF expression and vessel formation, and thereby weakened synovial fibrosis. The slight articular cartilage injury histopathology and regular gait characteristics of affected knees underpinned the miR-29a harmonization of joint microenvironment homeostasis. Accumulating evidence has revealed that VEGF signaling component interruption alleviates OA pathogenesis in terms of delayed synovitis, cartilage breakdown, subchondral bone damage, and joint pain^42, 43^. This study revealed an emerging VEGF inhibition strategy by administering miR-29a, which preserved the synovium integrity for prevention from OA.

Synovitis is correlated with the incidence of end-stage OA knees^44^ and asymptomatic OA knees^45^. Analyses of current study revealed that miR-29a protected from synovial damage in OA joints. The biological actions of miR-29a to synovial tissue metabolism in the development of asymptomatic OA is worthy of investigation. Moreover, synovitis occurs in the development of collagenase-^46^, destabilized medial meniscus-^47^ and mechanical stress-induced^26^ OA knees in mice. miR-29a deficiency modulates inflammatory reactions in mice with collagen-induced arthritic joints^48^. While the biological actions of miR-29a to surgical- or loading-mediated synovium deterioration warrants further investigation, the analyses of miR-29a alleviation of OA synovitis in collagenase-affected knees explained the phenomenon of miR-29a deficiency exacerbation of synovial integrity existed in patients with end-stage OA. The possibility that miR-29a signaling may directly or indirectly disrupt expressions of fibrotic matrices or inflammation regulators in OA synovial fibrosis cannot be discounted. miR-29a may directly affect the biological function of vascular endothelial cells, altering neovessel formation in synovial remodeling. Low angiogenic activities in HUVECs transfected with miR-29a precursor sustained the postulation of miR-29a actions to angiogenesis in the synovial microenvironment. Collective analyses revealed that injured synovial tissues responded to miR-29a signaling by modulating several biological activities. Simultaneous reduction in expressions of several deleterious factors e.g. IL-1β, MMPs and SDF-1, explained the contribution of miR-29a signaling to maintain synovial tissue metabolism.

**Figure 8:**
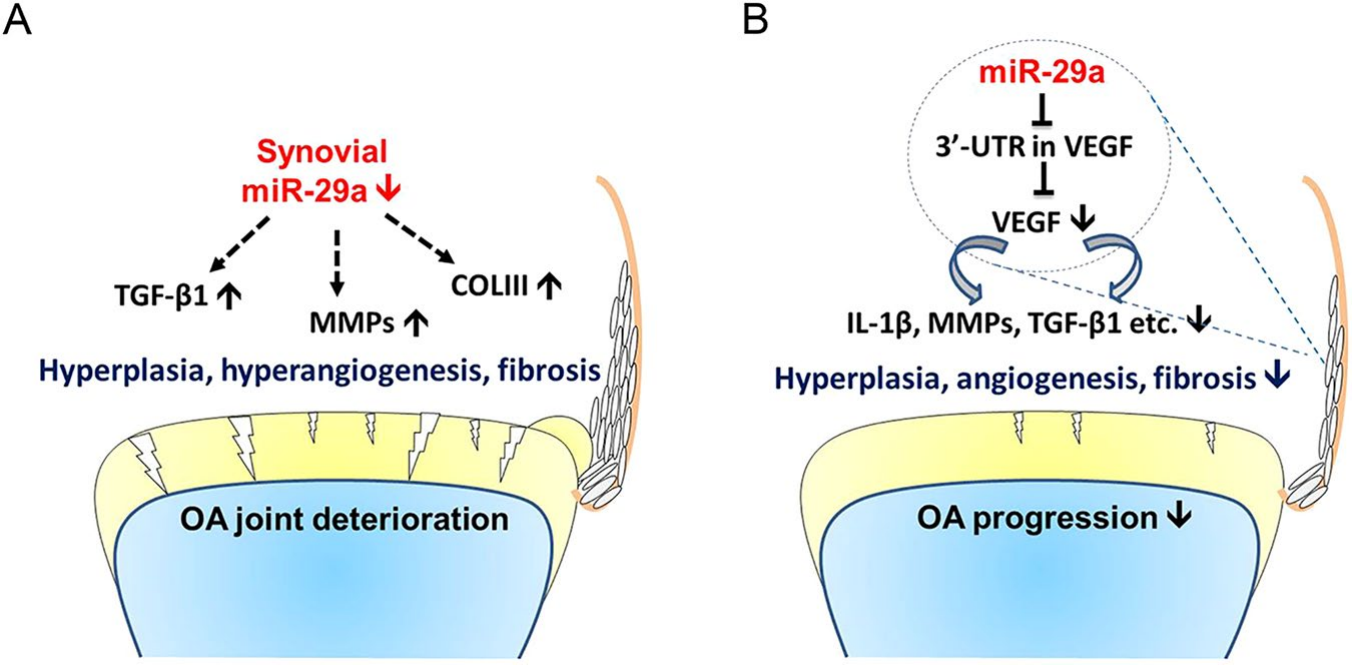
Scheme of miR-29a regulation of synovitis during OA knees. (**A**) miR-29a deficiency worsens synovial remodeling in terms of hyperplasia, hyperangiogenesis, and fibrosis in OA joints. (**B**) miR-29a mitigates synovitis through a direct action to the 3′-UTR of VEGF that reduced VEGF secretion and thereby protects from OA pathogenesis.

Taken together, this study reveals the inhibitory actions of miR-29a to synovitis. miR-29a deficiency accelerates fibrotic, inflammatory, and proteolytic factors in synovial fibroblasts from end-stage OA knees (Fig. 8A). Enhancement of miR-29a function shields from excessive fibrotic matrix deposition through interference in VEGF-mediated angiogenesis, an effect that fends off the occurrence of OA joints (Fig. 8B). These robust investigations also highlight a miR-29a therapeutic strategy for counteraction of OA joint development.

## Materials and Methods

### Clinical specimens

This study and experimental protocols were performed in compliance with the ethical guidelines and approved by the Institutional Review Board of Chang Gung Memorial Hospital (IRB approval No 104-5248B). All patients were well-informed, and written consent was obtained. In the OA group, 20 patients with end-stage OA knee (12 females and 8 males; 69.4 ± 1.9 years old) who required total knee replacement were recruited, and synovial tissue within the affected joints was biopsied. In the non-OA group, tissue was harvested from 8 participants (6 females and 2 males; 66.8 ± 1.9 years old) with femoral neck fracture who required hemiarthroplasty.

### Synovial fibroblast cultures and transfection

Fresh synovial tissue was subjected to enzyme digestion (0.03% trypsin and 0.3% collagenase), filtration through sterile 20-μm sieves, and isolation of synovial fibroblasts. Cell cultures were incubated in DMEM and 10% fetal bovine serum until 80% confluence was reached, as previously described^49^. Synovial fibroblasts (1 × 10^5^ cells/well, 6-well plates) were transfected with 30 nM miR-29a precursor (Ambion Pre-miR™; Life Technologies), antisense oligonucleotide (Ambion Anti-miR™), and scramble control using Lipofectamin RNAiMAX (Life Technologies), according to the manufacturer’s instructions.

### Detection of miR-29a binding to 3′-UTR of VEGF

The sequence of the VEGF 3′-UTR within the putative miR-29a binding site was cloned and inserted into pCRII-TOPO II luciferase receptor vectors (Invitrogen) using specific primers (Supplementary Table 1). A single-base mutant (5′-AUUUTUUU-3′) of the VEGF 3′-UTR (5′-AUUUAUUU-3′) (NM_001025367) was constructed. Reporter vectors for the intact VEGF 3′-UTR, mutated VEGF 3′-UTR, and renilla luciferase were transfected into subconfluent synovial fibroblasts. The cell cultures were then transfected with 30 nM miR-29a, antisense oligonucleotide, and scramble control. Luciferase reactions were detected using dual luciferase detection kits (Promega), normalized to the renilla luciferase activity and expressed as arbitrary luminescence units (RLUs).

### ELISA

Aliquots of synovial fibroblasts (1 × 10^5^ cells/well, 24-well plate) transfected with miR-29a, antisense oligonucleotide, and scramble control were incubated in basal medium. After incubation for 24 hours, culture media of synovial fibroblasts transfected with miR-29a, antisense oligonucleotide, and scramble control were harvested and centrifuged. The VEGF levels in the culture supernatants were quantified using VEGF ELISA kits (R&D Systems).

### Assessment of tube formation in umbilical vessel endothelial cells

*In vitro* angiogenic capacity was analyzed using BD BioCoat^TM^ angiogenesis kits (BD Biosciences), according to the manufacturer’s instructions. Aliquots of human umbilical vessel endothelial cells (2 × 10^4^ cells/well, 96-well plate; American Type Cell Collection) were incubated in a mixture containing equal volumes of basal medium and culture supernatants harvested from synovial fibroblasts in a 37 °C incubator for 6 hours. In some experiments, basal medium was mixed with 5 ng/ml VEGF recombinant protein (R&D Systems). The numbers of tube and vessel ring were counted. Six fields in each well and 6 wells of each group were randomly selected for quantification.

### miR-29a transgenic mice

Animal use protocols were in compliance with the guidelines and approved by the Institutional Animal Care and Use Committee (IACUC No. 2013053001) of the Kaohsiung Chang Gung Memorial Hospital. The FVB mice that overexpressed phosphoglycerate kinase (PGK) promoter control of miR-29a (FVB/TNar-Tg-miR29a/PGK; miR-29aTg) were bred and genotyped using PCR and primers (forward: 5′-GAGGATCCCCTCAAGGATACC AAGGGATGAAT-3′; reverse, 5′-CTTCTAGAAGGA GTGTTTCTAGGTTCCGTCA-3′), as previously described^50^. Male littermates that did not express miR-29a construct were designated as wild-type mice. In some experiments, 7-day-old neonatal miR-29aTg (n = 8) and wild-type mice (n = 8) were euthanatized and subjected to dissecting cartilage in knee joints^51^. After collagenase I digestion of the cartilage specimens, 10^6^ chondrocytes were incubated in 10-cm dishes containing DMEM and 10% FBS for 24 hours, as previously described^51^. The chondrocyte cultures were subjected to extracting total RNA for RT-PCR.

### Collagenase-induced OA knee

Thirty-two 3-month-old male miR-29aTg mice and 32 wild-type mice were evenly divided into the OA and control groups, respectively. A mixture containing 1000 units/ml collagenase was prepared with sterile normal saline. After anesthetized with inhale isofurane, the animal’s left knees were subjected to injection of 10 μl collagenase (10 units) and normal saline via an intra-articular needle, as previously described^52^. Animals were euthanatized with an overdose of Zoletil and Xylazine at 8 weeks after injection; the affected limbs were dissected and cleared off skin and muscle. Eight specimens from each group were randomly selected for histomorphometry and 8 specimens were subjected to RT-PCR analysis.

### Exogenous miR-29a administration

Lentivirus-shuttled pMIF-cGFP-zeo expression vectors (System Biosciences) that coded the miR-29a precursor were constructed and co-transfected with pPACKF1 vectors into 293 T cells. After titration using LentiX qRT-PCR Titration kits (Clontech), each 10-μl suspension containing 1 × 10^10^ infectious units of lentivirus particles was prepared for injection into affected joints. Sixty-four male FVB/N mice were divided into control, OA, OA-miR-29a, and OA-mock groups. Injured joints (2 weeks after collagenase aggravation) were administered saline, lentivirus-shuttled miR-29a, and mock through an intra-articular injection. After euthanasia, 16 affected joints in each group were dissected for study at 8 weeks after collagenase injection. 8 injured joints were subjected to total RNA extraction and 8 were harvested for histologic analysis.

### Gait analysis

A CatWalk system with a walkway that was monitored by sensitive cameras and sensors (Noldus Information Technology) was used to characterize the gait profiles of each animal. Walking patterns were simultaneously recorded as animals passed through the walkway. Each animal was evaluated at least 4 times. CatWalk software 9.1 and CatWalk XT’s Automatic Footprint Classification software were used to compute footprint patterns, stride length (mm) swing time (s), swing speed (mm/s), stand time (s), paw contact, area (mm^2^), and intensity (arbitrary unit).

### Histomorphometry

Sagittal sections of decalcified specimens were subjected to hematoxylin-eosin, Safranin O, and Masson’s trichrome staining (Sigma-Aldrich) for assessment of joint morphology and fibrosis tissues. The synovial and cartilage morphologies of 5 sections obtained from affected joints, spanning 400 mm for each specimen, were microscopically evaluated (20× object lens). The average synovial thickness at 3 points within the synovial membrane in each field was measured. The number of vessels within the synovial tissue in each field was analyzed using a Zeiss microscope and image analysis software. Areas of fibrosis tissue and total tissue in each field were measured and expressed as the percentage of fibrosis tissue area/total tissue area. Three fields (20× object lens) in each section and 24 sections from 8 mice were selected for analysis.

### Immunohistochemistry and *in situ* hybridization

Designated immunostaining in sections was detected by ED-1, VEGF, and HIF-1α antibody (Abcam), using horseradish peroxidase-conjugated IgG (BioGenex Laboratories) as the secondary antibody. Sections probed with isotype IgG were used as negative controls. In some experiments, sections were subjected to prehybridization and hybridization using IsHyb *In Situ* Hybridization kits (Biochain) and reacted with miR-29a probes that were labeled with digoxigenin (Applied Biosystems). miR-29a expression in sections was detected using horseradish peroxidase-conjugated digoxigenin antibody (Roche), according to the manufacturer’s instructions. Number of cells positive for immunostaining and cells positive for hematoxylin stain within synovial tissue in each field (40× object lens) was counted. Immunoreactivity of designated molecules was calculated as the percentage of number of immunostained cells/number of hematoxylin-stained cells. Three fields in each section and 24 sections from 8 mice were selected for analysis.

### RT-quantitative PCR

All specimens were prepared in RNase-free conditions. Microdissection of synovial tissue was performed under a Ziess DPMI PICO dissection microscope, as previously described^4^. In brief, the synovial membrane on femurs within affected joints was exposed after arthrotomy of the tibiae end and transection of the patellar tendon. The synovial membrane of interest was dissected. After reverse transcription of 1 μg total RNA that was extracted using Qiagen RNA extraction kits, aliquots of RT products were mixed with specific primers (Supplementary Table 1) and 2× TaqMan^®^ Universal PCR Master Mix (Applied Biosystems). An ABI 7900 Sequence Detection System (Applied Biosystems) was used for PCR, with reactions performed at 95 °C for 20 s, then 40 cycles at 95 °C for 3 s and 60 °C for 20 s, with computation of the intensity threshold (Ct). Amplification specificity was indicated by a dissociation curve exhibiting a single peak during the PCR reaction. Calculation of mRNA expression was according to the equation 2^−ΔCt^, where ∆Ct = Ct_gene_ − Ct_β-actin_.

### Statistical analysis

Significant differences (P < 0.05) between clinical specimens from OA and non-OA groups; primary chondrocytes from the miR-29aTg and wild-type mice were analyzed using the Wilcoxon test. Normal distribution of analyses of synovial fibroblasts, the miR-29aTg mice and wild-type mice with and without intra-articular collagenase injection were verified using Sharpiro-Wilk tests. Significant differences (P < 0.05) among 3 or more groups were further analyzed using parametric analysis of variance (ANOVA) and the Bonferroni post-hoc test.

**IRB Approval No.:** IRB approval No 104-5248B of Chang Gung Memorial Hospital.

**IACUC Approval No.:** IACUC No. 2013053001 of Chang Gung Memorial Hospital.

## Electronic supplementary material

Supplementary Table 1
