# Supplementary material for: MicroRNA-29a Counteracts Synovitis in Knee Osteoarthritis Pathogenesis by Targeting VEGF
Source: Sci Rep. 2017 Jun 15;7:3584. doi: 10.1038/s41598-017-03616-w (PMC5472675; doi:10.1038/s41598-017-03616-w)
Supplement: Supplementary file 1 — Supplementary Table 1 [file 41598_2017_3616_MOESM1_ESM.doc]

MicroRNA-29a Counteracts Synovitis in Knee Osteoarthritis Pathogenesis by Targeting VEGF

Jih-Yang Ko, Mel S. Lee, Wei-Shiung Lian, Wen-Tsan Weng, Yi-Chih Sun, Yu-Shan Chen, Feng-Sheng Wang

**Supplementary Table 1**

**Sequences of primers for RT-quantitative PCR analysis of mRNA expression**

| **Primers for RT-quantitative PCR assays of human specimens** | |
| --- | --- |
| Collagen III | Forward: 5’-GCAGGGAACAACTTGATGGT-3’  Reverse: 5’-GGGAACATCCTCCTTCAACA-3’ |
| TGF-β1 | Forward: 5’-CGACTACTACGCCAAGGAGG-3’  Reverse: 5’-CACGTGCTGCTCCACTTTTA-3’ |
| PLOD2 | Forward: 5’-ATCATTGCTCCTCTTGTAACTC-3’  Reverse: 5’-CATTCCATACTCCTACTCTATTCC-3’ |
| MMP3 | Forward: 5’-CCACTCTATCACTCACTCAC-3’  Reverse: 5’-CAGCATCAAAGGACAAAGC-3’ |
| ADAMTS5 | Forward: 5’-GCTGTGCTGTGATTGAAGAC-3’  Reverse: 5’-TGTGATGGTGGCTGAAGTG-3’ |
| MMP9 | Forward: 5’-CATCGTCATCCAGTTTGGTG-3’  Reverse: 5’-AGGGACCACAACTCGTCATC-3’ |
| VEGF | Forward: 5’-ATGCAATGTAACCGTAAGC-3’  Reverse: 5’-CGTACCTAATGATACGTTACA-3’ |
| β-Actin | Forward: 5’-GTAACCCGTTGAACCCCATT-3’  Reverse: 5’-CCATCCAATCGGTAGTAGCG-3’ |
| **Primers for RT-quantitative PCR assays of mouse specimens** | |
| Collagen III | Forward: 5’-GTTCTAGAGGATGGCTGTACTAAACACA-3’  Reverse: 5’-TTGCCTTGCGTGTTTGATATC-3’ |
| TGF-β1 | Forward: 5’-GACTTTTCCGCTGCTACTGC-3’  Reverse: 5’-AATAGGGGCGTCTGAGGAAC-3’ |
| PLOD2 | Forward: 5’-TCTGAACGAGCCTGTGATG-3’  Reverse: 5’-GTGTGACTGGAGCAATGAAC-3’ |
| IL-1β | Forward, 5’-CAGGCAGGCAGTATCACTCA-3’;  Reverse: 5’-AGCTCATATGGGTCCGACAG-3’ |
| MMP9 | Forward: 5’-CCAGATGATGGGAGAGAAGC-3’  Reverse:5’- GGCCTTTGAAGGTTTGGAAT-3’ |
| ADAMTS5 | Forward: 5’-CCTCTTGGTGGCTGACTC-3’  Reverse: 5’-CGGATGTGGTTCTCAATGC-3’ |
| VEGF | Forward: 5’-CTCTTCGAGGAGCACTTTGG-3’  Reverse: 5’-TGTATGTGGGTGGGTGTGTC-3’ |
| VEGF-R1 | Forward: 5’-ACGGAAACCTGTCCAACTA-3’  Reverse: 5’-GGTTCCAGGCTCTCTTTCTT-3’ |
| VEGF-R2 | Forward: 5’-GCGGAGACGCTCTTCATAATA-3’  Reverse: 5’-GACAAGAAGGAGCCAGAAGAA-3’ |
| SDF1 | Forward: 5’-TGAGGATAGCAGGCTGGACT-3’  Reverse: 5’-GTTCTTCCCAGTGTCCGGTA-3’ |
| TIMP1 | Forward: 5'-CTCTGGCATCTGGCATCC-3’  Reverse: 5’-GCTGGTATAAGGTGGTCTCG-3' |
| ADAM12 | Forward: 5’-AGAAACACACAAAGACAACAGAAG-3’  Reverse: 5’-AAGGCACCATCGGCATCC-3’ |
| MMP3 | Forward: 5’-TGACGATGATGAACGATGG-3’  Reverse: 5’-TGGAGGACTTGTAGACTGG-3’ |
| β-actin | Forward: 5’-GACGGCCAGGTCATCACTAT-3’  Reverse: 3’-CTTCTGCATCCTGTCAGCAA-5’ |
